# In the Hands of a Robot, From the Operating Room to the Courtroom: The Medicolegal Considerations of Robotic Surgery

**DOI:** 10.7759/cureus.43634

**Published:** 2023-08-17

**Authors:** Satvik N Pai, Madhan Jeyaraman, Naveen Jeyaraman, Arulkumar Nallakumarasamy, Sankalp Yadav

**Affiliations:** 1 Orthopaedic Surgery, Hospital for Orthopedics, Sports Medicine, Arthritis, and Trauma (HOSMAT) Hospital, Bangalore, IND; 2 Orthopaedics, ACS Medical College and Hospital, Dr. MGR Educational and Research Institute, Chennai, IND; 3 Medicine, Shri Madan Lal Khurana Chest Clinic, New Delhi, IND

**Keywords:** litigation, court, law, medicolegal, robotics

## Abstract

Robotic surgery has rapidly evolved as a groundbreaking field in medicine, revolutionizing surgical practices across various specialties. Despite its numerous benefits, the adoption of robotic surgery faces significant medicolegal challenges. This article delves into the underexplored legal implications of robotic surgery and identifies three distinct medicolegal problems. First, the lack of standardized training and credentialing for robotic surgery poses potential risks to patient safety and surgeon competence. Second, informed consent processes require additional considerations to ensure patients are fully aware of the technology's capabilities and potential risks. Finally, the issue of legal liability becomes complex due to the involvement of multiple stakeholders in the functioning of robotic systems. The article highlights the need for comprehensive guidelines, regulations, and training programs to navigate the medicolegal aspects of robotic surgery effectively, thereby unlocking its full potential for the future..

## Introduction and background

Robotic surgery is a fast-growing field of medicine that is rapidly changing the surgical landscape. Though robotics has been used in other fields since the early 19th century, it made inroads in the field of medicine only about four decades ago [1]. Since then, however, the field has experienced remarkable growth and transformation. Over this time, there have been major advancements in the technology and application of these systems and devices [2]. Robotic surgery is currently being used widely in almost all branches of surgery, including neurosurgery [3], spine surgery [4], endocrine surgery [5], oncosurgery [6,7], orthopedic surgery [8], ophthalmology [9], gynecology [10], plastic surgery [11], pediatric surgery [12], thoracic surgery, and dentistry [13]. Yet, it still possesses several novel problems and dilemmas that need to be figured out conclusively. Today, robotic surgery stands at a crossroads, and the direction it proceeds in will be a significant factor in determining what surgery and the medical field can accomplish in the future.

Robotic surgery offers several benefits over conventional nonrobotic surgery that make it very promising. Robotic systems offer a range of advantages, including the ability to provide virtual information, superior precision in spatial resolution and geometric accuracy, improved dexterity, quicker maneuverability, and the ability to operate without fatigue, ensuring consistent and steady motion [14]. It also provides the ability for surgeries to be less invasive [15]. Robotic surgery can also significantly decrease blood loss and postoperative recovery periods, such as the length of the hospital stay. The ultimate benefit of all these factors is that they can improve patient outcomes [16,17]. Despite countering certain human limitations, robotic surgery can also involve several complications [18]. When robotic surgeries were first introduced, the primary constraints were the lack of availability and training. While those have been countered to some extent over the past few decades, certain flaws still exist [19,20]. Robotic malfunctions, though rare, can occur and may necessitate a change in the planned surgical procedure [21]. Mechanical failure or malfunction can rarely even cause patient injury [22]. The higher costs involved remain an impediment to its adoption, especially in developing countries [23]. Some believe that robotic surgery also poses a danger to surgeons’ capabilities, with the risk of a surgeon being overshadowed by a machine that's faster, stronger, more knowledgeable, and more efficient.

While all of the aforementioned factors will continue to evolve with advancements in technology, there is a unique aspect of robotic surgery that is often ignored that we want to address. This pertains to the medicolegal aspects of robotic surgery. The ethical and legal implications of its use have yet to be explored and understood, even by medical professionals using it [24]. We delve deep into the topic to understand what the current practices and regulations reveal. We also discuss certain perspectives on it and foresee some unique challenges ahead. We have extensively reviewed the available literature and identified three distinctive medicolegal problems associated with robotic surgery, each of which we elaborate on in the following sections.

## Review

Training and credentialing in robotic surgery

Despite robotic surgery being designed to decrease human error, there is no denying that the safety of robotic surgery still depends on the skill and training of the surgeon operating the robot [25]. Utilizing robots necessitates additional knowledge of the working of the robot and its method of use, apart from the surgical and anatomical knowledge of the procedure planned. Acquiring specific training in robotic surgery has been shown to decrease surgical errors significantly [26]. Training in robotic surgery, though, is still in its nascent stages, and credentialing for the same is yet to be formalized in most centers and regions [27]. With better exposure and opportunities, robotic surgery training is available but still requires the introduction of more structured training programs [28]. Most resident training programs in India are severely lacking in exposure to robotic surgery, necessitating fellowship programs [29]. Beliefs that laparoscopic surgical skills and training are easily transferable to robotic surgery are a common misconception [30]. A recent focus has been to incorporate robotic surgery training as a part of resident training programs [31]. The ideology behind this is that robotic surgery is soon becoming an integral part of most surgical subspecialties, so it may be a good idea to train surgeons in robotics from an early part of their careers. This would also increase exposure, adoption, and expertise in robotic surgery. However, this approach may not be as simple as it sounds at first, as it can be counterproductive as well [32]. Several studies have shown that resident training programs that included a significant amount of robotic surgery training demonstrated dissatisfaction among residents and a perception among many that the introduction and dependence on robotic surgery in resident training hamper their learning and skill concerning open conventional surgery [29,33]. Recent discussions and conferences on this subject have garnered some useful expert consensus recommendations and program curricula that can be utilized for future policymaking [34,35]. When it comes to credentialing for robotic surgery, there remains an overwhelming amount of ambiguity. Current credentialing practices are inadequate and nonstandardized [36]. A lack of clear credentialing can increase the liability risk for surgeons performing robotic surgery [37]. Unfortunately, no dedicated curriculum or credentialing system exists for robotic surgery in most countries, including India [38]. It is about time regulatory bodies recognized this issue and introduced more standardized systems or regulations. Credentialing model recommendations [39] can be reviewed, and a model tailored to each country needs to be developed. The implementation of guidelines and proctoring recommendations is necessary to protect surgeons, institutions, manufacturers, and, above all, the patients who are undergoing robotic surgery [40].

Informed consent for robotic surgery

Informed consent is an important process for developing trust and transparency between the surgeon and the patient. It also acts as a medicolegal document that often becomes the crux of many malpractice claims [41]. It is mandated as per the law that patients be informed about the basic steps and course of the surgical procedure they are proposed to undergo [42]. Hence, it is mandatory that, if a patient is planning to undergo robotic surgery, their consent for the use of the robot be obtained beforehand. This need is further amplified by the fact that most patients’ knowledge about robotic surgery is limited [43]. They may also have certain misconceptions that need to be cleared before the procedure [44]. A very common misconception that patients have when they are told they are to undergo robotic surgery is that they assume it to be a completely automated machine that will be operating without a surgeon or human interference. This is rarely the case and needs to be clarified at the time of decision-making for the surgery. Setting realistic expectations for surgeries has been shown to decrease the incidence of lawsuits following surgery [45].

When a robotic surgery is planned, in addition to the normal constituents of informed consent for a surgical procedure, certain additional aspects of the surgery need to be discussed. The risk of a robotic malfunction, though minimal, should be disclosed [46]. The exact steps in the surgery that would be robot-assisted also need to be communicated [47]. The advantages, risks, and alternative surgical options to robotic surgery also need to be informed. Some authors recommend disclosing the surgeon’s experience and training in robotic surgery and the number of robotic procedures performed prior by the department, but this is not a mandatory requisite [46]. The informed consent for robotic surgery need not necessarily require an entirely different consent form from nonrobotic surgery, but the informed consent taken for the surgery will have to mention the involvement of the robot for the surgery and the risks involved with it.

Legal liability

Coming to probably the most important section of this topic, on the legal liability and accountability for robotic surgery, we must understand that the legal stance on this is by no means a simple issue. There are various stakeholders involved, and balancing this legally and attributing responsibility is quite a challenge. There is no clear consensus on the legal standing of this matter, and the laws and guidelines about it are still evolving [48]. The surgeon, the hospital, and the manufacturer of the machine are all involved in the functioning or application of the machine and hence have some share of the legal responsibility. It also means that when faced with litigation, this is a recipe for a lot of finger-pointing between the parties [49]. Should certain surgeons harbor the misconception that robotic surgery might mitigate their legal responsibility for any adverse outcomes, we urgently seek to dispel this erroneous belief. The adoption of robotic surgery does not, in any way, exonerate the surgeon from their legal accountability [50]. This is because courts of law to date have predominantly seen the use of robots as tools of assistance to surgeons, the utility of which helps surgeons, but still expected the surgeons to be able to use their discretion on the proposed actions of robots and provide a level of human check on any proposed actions of the robots [51].

With the relatively limited availability of legal literature and verdicts on this matter, there may be knowledge to be obtained from another area where this conundrum has surfaced: dealing with autonomously driven cars. Self-driven cars, as they are often called, predominantly operate autonomously but are required to be monitored by a human being. They would therefore share many similarities with robotic surgery, one of which is that a mishap can be catastrophic and life-threatening [26]. It also created the same problem of determining culpability in such situations. Even with automated vehicles, it is noted that a shared responsibility exists. While the law has tried to be more control-specific, based on the level of autonomy and control of the actions leading to the accident, there is no denying that the driver shares some of the legal responsibility [52]. An analysis of the legal proceedings in cases involving crashes involving autonomous vehicles has shown that culpability does vary on a case-by-case basis. If the crash involved fully autonomous vehicles, the human users were held less liable, and a majority of the responsibility was to be shouldered by the manufacturer and the government department that issued the manufacturing licenses [53]. But there have been instances where the driver has been attributed to blame despite having limited ability to avoid the accident [54]. Experts have analyzed these cases and also noted that there is a certain amount of bias when it comes to rewarding compensation for victims in such cases. According to what is called ‘blame attribution asymmetry bias’, humans are prejudiced to judge these cases more harshly, ascribe more blame to the automation and its creators, and be inclined to award more compensation to the victims than in a similar case where no autonomous vehicle was involved [55]. This bias can impact future policies and deter the adoption of such technologies.

The summary of the analysis of legal liability when it comes to autonomously driven vehicles is that the jury is still out and culpability is being determined on a case-by-case basis. We can expect cases involving robotic surgery to be decided based on similar legal principles, considering the parallels, but one must remember that surgery would still be seen a little differently. A surgeon is considered to have more control, impact, and discretionary power than a user of an autonomous vehicle. Even with the invention of certain fully autonomous surgical robotic systems, the surgeon has been ascribed some of the blame for the adverse results of such robotic systems [56]. Surgeons may thus end up being the convenient bearers of responsibility in this complex legal scenario prevailing currently. The current laws do not appear to be directly applicable to these novel technological inventions, and new legal frameworks have to be developed to be adept in such cases [57]. All the aforementioned factors have to be taken into consideration, and regulations based on the different levels of autonomy and context of use have to be introduced [58]. This would require meticulously drafted guidelines, framed after understanding the workings of machine systems and the types of malfunctions.

Despite the immense technological advances in robotic surgery, there is still an overwhelming lack of clarity on the surgeon’s legal responsibility for surgical robot malfunctions [59]. Questions remain on the liability of the manufacturer as well as whether the robot has passed the required quality checks and obtained the necessary government licenses for use. Making laws/guidelines regarding the use of robotics in surgery is not an easy process either. Apart from dealing with the various conundrums discussed earlier, every country has to take into consideration its own legal principles, and social factors and frame its own law which is suitable and in keeping with the other laws of the country. Directly adopting a law from another country or replicating it, would not be advisable. An additional pragmatic factor in assessing liability is the challenge of confidently distinguishing whether the negative outcomes stem from a malfunction in the robotic system or incorrect utilization by the surgeon. Consequently, as we venture into the relatively unexplored realm of sophisticated robotic surgeries, surgeons are urged to navigate with caution and diligence rather than hastily disregarding potential risks.

The future of robotic surgery

In the forthcoming decades, we can anticipate a significant surge in the realm of robotic surgery. Advancements in technology, refinements in technique, and increased acceptance globally are projected to propel its growth exponentially [60]. The utility and indications are expanding and are likely to become an integral part of surgical practice worldwide in the future [61]. Several new platforms and machine systems are being invented, and more are in the pipeline for the future [62]. The level of precision and ability of robotic systems will increase further with breakthroughs like tremor filtering, enhanced three-dimensional (3D) high-definition (HD) vision, fully wristed instrumentation with several degrees of freedom, etc. The amalgamation of robotic surgery and artificial intelligence is set to revolutionize the surgical field again, as it may lead us into the age of technology-driven surgery [61,63]. We believe that the potential obstacles to this growth trajectory will not stem from technology-related issues. Instead, the challenges are likely to arise from the non-surgical aspects of the technology. The legal problems it might run into can be a discouragement for some surgeons, as can the ethical aspects of it for others [64,65]. Manufacturers should not only focus on increasing autonomy but also prioritize collaborative robotic surgery [66] since the rate of adoption of robotic surgery is not going to be based solely on surgical outcomes but is also going to be influenced by surgeon satisfaction [67]. To decrease adverse results or accidents, manufacturers should release manuals for problem-solving for each machine, and surgeons should not hesitate to use these as they familiarize themselves with these new entrants in our operation theaters [68]. As the machinery landscape transforms, our learning modalities, training methodologies, and credentialing systems must adapt concurrently [69-71]. Correspondingly, our legal framework must also evolve in tandem with these advancements. It will be a continuous process of improvement to further decrease the possibility of adverse outcomes or machine malfunctions in robotic surgery [72-74]. Another area of progress required is in ensuring that these systems are secure and protected from cyber attacks or misuse [75]. This proactive approach is critical to secure a future where these advancements are not impeded by legal hurdles.

## Conclusions

While robotic surgery has been growing in technology, technique, and popularity, one aspect that is still shrouded in ambiguity is its legal aspect. There is still a lack of clear laws and legal guidelines on the legal liability of surgeons and manufacturers. There is also a glaring deficiency in organized training and credentialing. This makes us believe that the moral, ethical, and legal aspects of robotic surgery may end up playing a bigger part in determining the future of robotic surgery than most people would suspect and desire.
